# Emergence and Molecular Characterization of an Avian Hepatitis E Virus From Donglan Black Chicken in Southern China

**DOI:** 10.3389/fvets.2022.901292

**Published:** 2022-05-26

**Authors:** Fumei Fu, Qiaomu Deng, Qiuhong Li, Weiyu Zhu, Jinhan Guo, Ping Wei

**Affiliations:** Institute for Poultry Science and Health, Guangxi University, Nanning, China

**Keywords:** avian hepatitis E virus, Bayesian analysis, genotype, Donglan Black chicken, transmission

## Abstract

Avian hepatitis E virus (HEV) is a major pathogen associated with hepatitis splenomegaly syndrome in chickens and has been reported in China. Phylogenetic trees, Bayesian analysis, positive selection sites screening, and recombination analysis were first used to comprehend the global avian HEVs. All the avian HEV strains, including a new isolate named GX20A1 got from Donglan Black chicken in Guangxi, China, were uniformly defined into four genotypes, and GX20A1, belongs to Genotype 3. The topology of the phylogenetic tree based on the sequences of a 339-bp fragment (coding the helicase) in open reading frame (ORF) 1 of the avian HEVs was consistent with that based on the full-genome sequence. The estimated evolution rate of avian HEVs is 2.73 × 10^−3^ substitution/site/year (95% confidence interval (*CI*): 8.01 × 10^−4^−4.91 × 10^−3^), and the estimated genetic diversity of the strains experienced a declining phase from 2010 to 2017 and stabilized after 2017. It was further found that the Genotype 3 HEVs, including isolates from Hungary and China, likely originated in the 1930s. Notably, GX20A1 was gathered in the same branch with a Genotype 3 Guangdong isolate CaHEV-GDSZ01, which appeared earlier than GX20A1. In addition, two positive selection sites were identified, one for each of ORF1 and ORF2. Overall, the study revealed that avian HEVs were uniformly defined into four genotypes, and a 339-bp fragment in ORF1 of the viral genome could be used for the classification. A Genotype 3 isolate GX20A1 was first found from Donglan Black chicken and most likely originated from Guangdong.

## Introduction

Avian hepatitis E virus (HEV) is the primary pathogen of big liver and spleen (BLS) disease or hepatitis-splenomegaly (HS) syndrome in chickens (1). Avian HEV mainly caused a decrease of egg production and an increase in mortality, enlargement of the liver and spleen, and bleeding and coagulation in the abdominal cavity (2). BLS was considered as an economically significant disease of broiler breeders and caused a drop in egg production (3). Avian HEV belongs to the family *Hepeviridae* and genus *Orthohepevirus* and species *Orthohepevirus* B, which is a single positive-stranded RNA virus (4). The avian HEV genome is about 6.6 kb in length, excluding the poly (A) tail (5). The avian HEV genome consists of a short 5' untranslated region (UTR), followed by three open reading frames (ORFs), and a 3'UTR (5). ORF1 is the longest ORF and encodes non-structural proteins of the virus, including several functional domains, such as the viral methyltransferase, helicase, and RNA-dependent RNA polymerase (6). ORF2 encodes the immunogenic capsid protein, and ORF3 encodes a phosphorylated protein (7). Genotyping of avian HEV is constantly being discovered and updated (8, 9), and a recent study divided avian HEV into five genotypes (10).

At present, avian HEV mainly infects broiler breeders and layers, the majority of which are subclinical infections (11, 12), and the main transmission mode is fecal-to-oral route. Besides detecting the target organ liver, avian HEV could be detected through the intestines and other organs (13). Previous studies also showed that the identification of avian HEV was mainly based on samples, such as liver, bile, and feces (2, 8, 14). In the past, avian HEV was mainly detected in the broiler breeders of White-chicken and the layers. However, two cases have recently emerged in local chicken breeds in China, but the sources and the transmissions are unknown (15, 16). The ongoing evolutionary and transmission routes of avian HEV remain poorly investigated. However, this information is essential for us to understand the origin and transmission routes of the global strains and to set up a preventive and control strategy.

In this study, an avian HEV isolate was detected in a Donglan Black chicken in December 2020 in Guangxi, China. This breed of chicken originated in Donglan County, has the nature characteristics of black feather, black skin, black meat and bone, and is mainly raised in the countryside. It is a unique local breed of chickens and has not been cross-bred with other breeds of chickens for commercial purposes (17). Based on the sequences of all the avian HEV isolates available in GenBank, the phylogenetic trees, Bayesian analysis, positive selection sites screening and recombination analysis were performed. These analyses may help us to better understand the molecular characteristics of the virus, evaluate the relationship between the circulating isolates from different sources, and grasp the transmission route of the virus in time, which will be beneficial to control the spread of the virus among different chicken breeds and geographical regions.

## Materials and Methods

### Case and Samples

An avian HEV isolate was obtained from a case that happened on a farm that raised the parent-stock of Donglan Black chickens, and some other breeds of Yellow-chicken are kept on the same farm in Guangxi, China, in 2020. Four Donglan chickens (20-week-old) suspected of avian HEV infection were sent to our laboratory. The farmer described a flock of 5,000 chickens with a daily mortality of 1–2% during the period from December 22 to 28, 2020. The clinical observations of the birds were loss of appetite and depression, polluted feathers around cloaca, and abnormally increased mortality in the flock. The birds were subjected to necropsy, and the significant observations included hemorrhage spots on the liver and enlargement of the liver and spleen. Samples from the liver and feces were aseptically collected and snap-frozen in liquid nitrogen immediately and stored at −80°C.

### Virus Identification

Total RNA was extracted from the liver and feces using a total RNA extraction kit (Axygen, USA). The extracted RNA was reverse transcribed into cDNA using the cDNA Reverse Transcription Kit (Takara, Dalian, China) and then PCR detection with two sets of primers (Supplementary Table 1) targeting the partial ORF2 capsid gene sequences of avian HEV was performed (12, 18). At the same time, total DNA was extracted from the liver tissue using the DNA extraction kit (TianGen, China), and the PCR detections for avian leukosis virus (ALV), Marek's disease virus (MDV), reticuloendotheliosis virus (REV), and fowl adenovirus serotype 4 (FAdV-4) were also performed, respectively, by using the primers (Supplementary Table 2) as our previous reports (19). The PCR products were sent to Shenzhen Huada Genomics Technology Service Co., Ltd. for sequencing. The generated avian HEV sequences were compared with other strains available in GenBank (https://www.ncbi.nlm.nih.gov/BLAST).

### Genome Sequencing

Based on the genome sequences of the CaHEV-GDSZ01 strain (MK050107), a total of nine sets of PCR primers (Supplementary Table 3) were designed and used to amplify the full-genome of avian HEV (20). The conditions for the PCR on the cDNA from the feces sample were an initial incubation at 95°C for 3 min, followed by 32 cycles, denaturation at 95°C for 15 s, annealing at 50°C for 15 s, extension at 72°C for 30 s, and a final extension at 72°C for 5 min. All PCR amplification products were analyzed on 1% agarose gel PCR products and then purified using the Gel Band Purification Kit (Genstar, China) and cloned into the pMD18-T vector (Takara, Dalian, China). The recombinant plasmids were transformed into *Escherichia coli* DH5α, and the positive clones were sequenced by the Shenzhen Huada Genomics Technology Service Co., Ltd. Three positive clones were sequenced for each fragment. The full-length cDNA sequence was assembled using the SeqMan program in the Lasergene (DNASTAR, Madison, WI).

### Phylogenetic Analysis

The most detailed genome sequences were obtained, including our isolate and 16 other isolates available in GenBank. An evolutionary tree was constructed using the complete/near-complete genome dataset (N1 = 17) by IQ-TREE v1.6.12 software (21). Using the BioEdit v7.2.6 and MEGA X software (22), 3 ORFs (ORF 1, 2, and 3) and the different length fragments of ORF1, were collated, including 6 datasets N2 (17), N3 (17), N4 (17), N5 [21, 177–2 922 nucleotides (nt)], N6 (25, 1,595–2,157 nt), and N7 (45, 2,584–2,922 nt). Details of all the sequences used in this study are shown in Supplementary Table 4. The evolutionary trees of 7 datasets were constructed. All trees were visualized in Figtree v1.4.3. According to the results of the phylogenetic trees, the dataset (N1 = 17) was used to estimate the mean inter-genotypes distances (nucleotide distances). The mean inter-genotypes distances were inferred using MEGA X. Analyses were conducted using the maximum composite likelihood (MCL) model (23).

### Bayesian Analyses

To determine the temporal structure of the dataset (N7 = 45), we performed a regression of root-to-tip genetic distance for the dataset using the TempEst v1.5.3 software (24). The Bayesian Markov chain Monte Carlo (MCMC) method implemented in BEAST v1.10.4 (25) in the CIPRES Science Gateway (https://www.phylo.org/) (26) was used to estimate the evolution rate and population dynamics. The HKY+G substitution model, an uncorrelated relaxed molecular clock model, and 3 demographic models (constant size, exponential growth, and Bayesian skyline) were selected (27). Each set was run for 200 million generations with every 20,000 cycles sampled to ensure the effective sample sizes (ESS) >200 using Tracer v1.7.1. The Bayesian skyline plot (BSP) inferred the changes in avian HEVs in an ESS between 1986 and 2020, plotted with Tracer v1.7.1. The maximum clade credibility (MCC) tree is generated using TreeAnnotator v1.10.4. The created MCC tree is visualized using Figtree v1.4.3.

To further explore the global distribution history in detail, the phylogeographic analysis was performed in BEAST v1.10.4. An asymmetric substitution model and an uncorrelated relaxed molecular clock model were selected. Other parameters are the same as those of the method described above.

### Selection Analysis

Selection pressure was analyzed using the Datamonkey web server (http://www.datamonkey.org). Based on the 4 methods, including fixed effects likelihood (*FEL, p* < 0.1), single likelihood ancestor counting (*SLAC, p* < 0.1), fast unconstrained Bayesian approximation (FUBAR, posterior probability (*pp*) > 0.9), and mixed-effects model of evolution (*MEME, p* < 0.1), the selection pressures on the ORF1, ORF2, and ORF3 were analyzed. A site is to be considered under positive selection when detected as such by at least three methods.

### Recombination Analysis

Recombination events in the complete genome of the avian HEVs were predicted using RDP5 v5.5.0 (28). Seven methods (RDP, BootScan, Geneconv, MaxChi, SiScan, Chimera, and 3Seq) were used to perform the recombination analysis of the virus genome. Recombination events predicted at least by the five algorithms with a value of *p* < 10^−3^ were considered valid.

## Results

### Observation of the Pathological Changes

**Figure 1:**
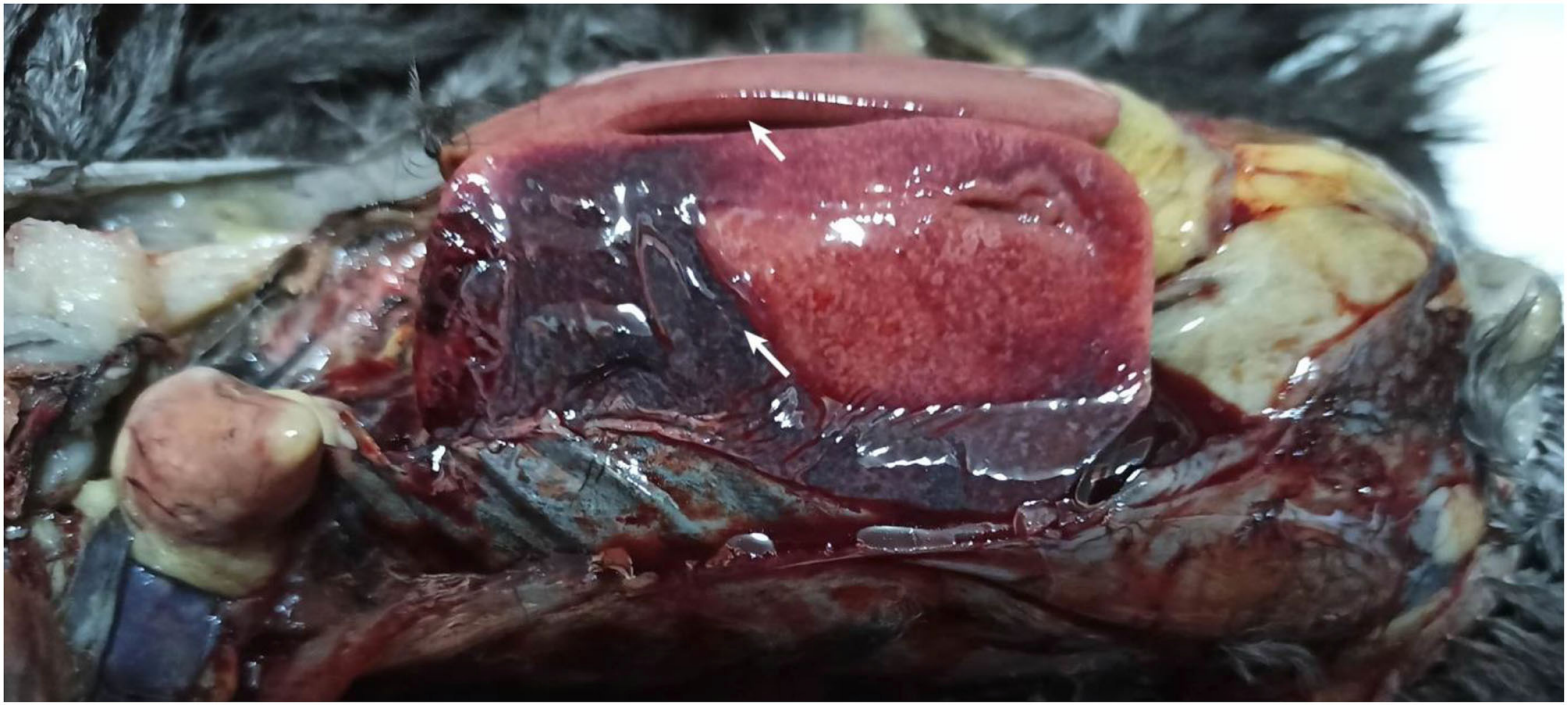
Inspection of the gross pathological changes of the necropsied Donglan Black chickens. Enlarged and clots of blood in the liver infected chickens (arrows).

Necropsy of birds mainly found enlargement of the liver and spleen, hemorrhagic spots, and a large area of blood clots on the liver surface (Figure 1). No obvious pathological changes were found in other organs/tissues.

### Identification of Avian HEV

Through simultaneous PCR detections on the liver and feces samples, the PCR results found that the feces samples got very specific target bands of the avian HEV (Supplementary Figure 1), while the liver samples also got the target bands plus the non-specific bands. An avian HEV was identified by sequence alignment with known GenBank sequence using the Basic Local Alignment Search Tool (BLAST) (Supplementary Figure 2). The positive sample from the feces was further used for the full-genome sequencing. The total DNA extracted from the samples was all negative for detecting the viruses MDV, ALV, REV, and FAdV-4.

### Genomic Characterization and Phylogenetic Analysis

The sequence of the near full-genome of the avian HEV isolate, named GX20A1, was obtained (GenBank accession number: OM747874) by a series of PCR amplification with the 9 sets of primers. The three major structural gene sequences are obtained, and only approximately 20–30 bases at the virus's 5' and 3' ends are not obtained. The obtained sequence is 6,633 nt in length, including partial 5'UTR (1–11 nt), entire 3'UTR (6,509–6,633 nt), and 3 complete ORFs: ORF1 (12–4,601 nt), ORF2 (4,688–6,508 nt), and ORF3 (4,635–4,898 nt).

The available avian HEV strains (N1 = 17) were uniformly defined into four genotypes based on the mean inter-genetic distances between the genotypes and ultrafast bootstrap value (Supplementary Tables 5, 6; Figure 1). Genotype 1 includes isolates from Australia and South Korea; Genotype 2 includes isolates from the United States, Australia, South Korea, and China; Genotype 3 includes isolates from Hungary and China; and a new genotype (Genotype 4) is currently found only in China.

**Figure 2:**
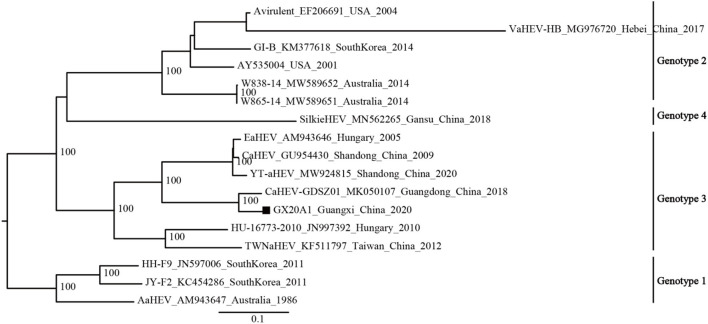
Phylogenetic tree was constructed by the near full-genome of avian hepatitis E virus (HEV) using IQ-TREE v1.6.12. A solid black square indicates the isolate in this study.

The phylogenetic analysis of the dataset (N1 = 17) shows that GX20A1 belongs to Genotype 3 and has the closest relationship with the Guangdong isolate CaHEV–GDSZ01, (ultrafast bootstrap value = 100) (Figure 2). Compared with the tree topology of the N1 dataset, the N2 dataset is consistent with it, while datasets N3 and N4 are inconsistent (29). The tree topology of datasets (N5, N6, and N7) is also consistent with the dataset N1. It reveals that the sequence of a 339-bp fragment dataset (45, 2,584–2,922 nt) in ORF1, could represent the whole genome sequences of avian HEV used for classification. The evolutionary tree of datasets (N2–N7), is shown in Supplementary Figure 3.

### Results of the Bayesian Analysis

**Figure 3:**
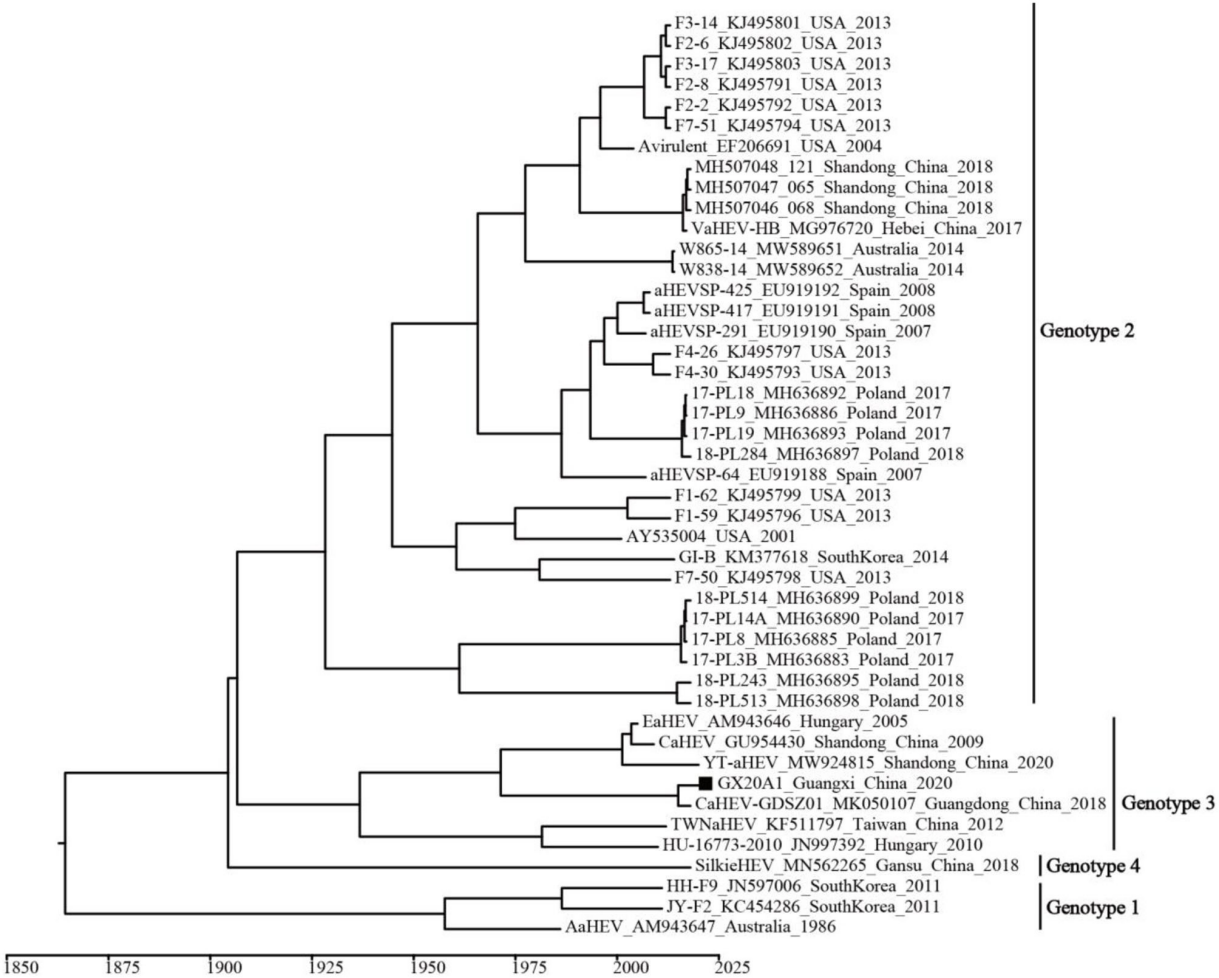
The maximum clade credibility (MCC) tree of avian HEV isolates based on the 339-bp fragment in open reading frame (ORF) 1 (N7 = 45). A solid black square indicates the isolate in this study.

**Figure 4:**
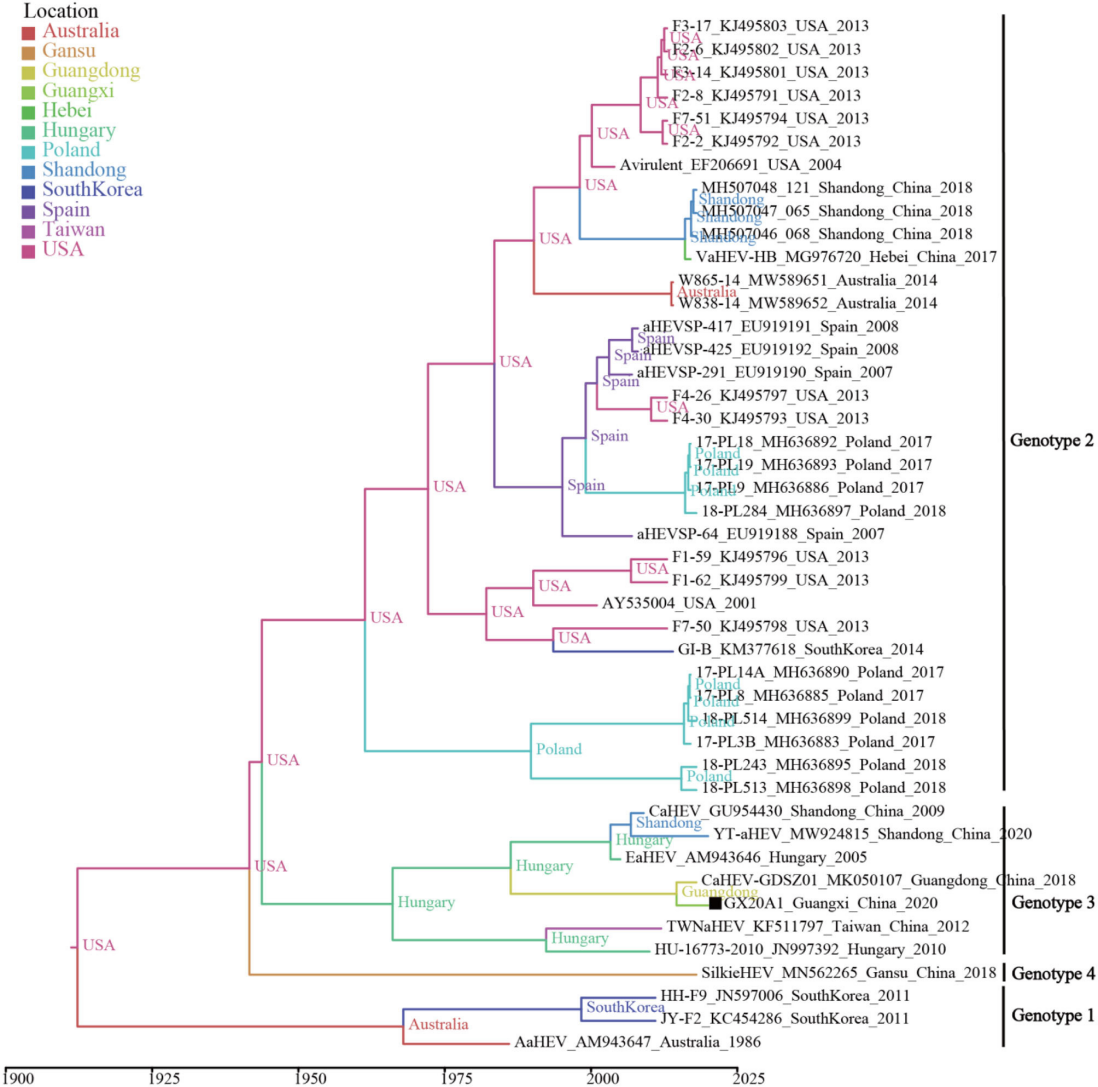
The MCC tree based on the 339-bp fragment in ORF1 (N7 = 45) of the avian HEV. The colors of the root and nodes correspond to the geographic regions. A solid black square indicates the isolate in this study.

A temporal signal was obtained (correlation coefficient = 0.650; *R*^2^ = 0.422). The estimated average evolution rate of the 339-bp fragment (N7 = 45) in ORF1, was 2.73 × 10^−3^ substitution/site/year (95% confidence interval (*CI*): 8.01 × 10^−4^–4.91 × 10^−3^). The MCC tree shows that all avian HEVs are divided into four genotypes, which is consistent with the genotypes identified by the ML tree (Figure 3). Genotype 3 includes isolates from Hungary and China, and originated in 1937 (95% *CI*: 1,831–1,989). As shown in Supplementary Figure 4, BSP reflects the relatively low genetic diversity of avian HEVs, and there is not enough evidence to support that avian HEVs are rapidly evolving. Phylogeographic infers that the United States strains may be at the root of the tree, and the dominant Genotype 3 isolates are likely to be spread from Hungary to China (i.e., Shandong, Guangdong, and Taiwan), as well as our isolate GX20A1 most likely originated from Guangdong (Figure 4).

### Results of the Selection Pressure Analysis

The results of the analysis with the four methods of SLAC, FEL, FUBAR, and MEME for ORF1, ORF2, and ORF3, respectively, are shown in Table 1. There are two positive selection sites, one in ORF1, and another in ORF2, and no positive selection site was found in the ORF3.

**Table 1:** The sites under positive selection in 3 open reading frames (ORFs).

**ORF**	**Positive selection sites by different models**			
	**MEME**	**FEL**	**FUBAR**	**SLAC**
ORF1	G246C, T309V, C570T, **A593P/V/T**, A638T/P, Q806S/E/T, A844R, T962P/S, N1065P, T1475A/D, V1519T	A557D/T/R/S/N, **A593P/V/T**	**A593P/V/T**	NA
ORF2	G5R, L23T, T163Y, T165H, V297S, V303T, **Q473T/M/P**, S539S	G5R, L23T, G27S/D, **Q473T/M/P**	**Q473T/M/P**	NA
ORF3	NA	NA	G16P/A	NA

*Only visualize p < 0.1 and pp > 0.9 and their close values; an avian HEV isolate (GenBank accession number: AM943647) was used as the reference and for aa-sequence alignment; and bold indicates sites predicted by three models to be under positive selection. NA: not available*.

### Recombination Analysis

By using the RDP5 v5.5.0 software, no reorganization event was found in isolate GX20A1.

## Discussions

In the study, an avian HEV strain GX20A1 isolated from Donglan Black chickens, a local chicken breed raised in a remote mountainous county and non-crossbred with other exotic breeds, but on a farm with multi-breeds of chickens. Compared with other breeds of Yellow-chicken, this breed is mainly used for preservation purposes and small-scale commercial production. However, the avian HEV has mainly the fecal-to-oral transmission route and isolate GX20A1, share the same common ancestor with Guangdong strain CaHEV-GDSZ01 by Bayesian analysis. Thus, it is speculated that the viruses carried by these exotic Yellow-chicken may have a common origin with the Guangdong strain, leading to the transmission of these viruses to the pure local breed chicken. Multi-breeds on one farm may pose a risk of spreading the virus from one to another. Generally, the infection of avian HEV has been found in many breeds of chickens (e.g., White-chicken and layers) (9, 14, 30), but two cases have been emerged in local chicken breeds in China recently (10, 15). Therefore, it is essential to detect and analyze the genome sequences of avian HEV isolates, which could provide some important information associated with the evolution and spread of the virus.

Previous studies reported that avian HEV could be detected in chicken feces, bile, liver, and gastrointestinal tissues samples (8, 31). Here the liver and feces samples in the diseased birds were collected to detect avian HEV by PCR. Compared with the amplification results of the liver samples, the feces sample got clear and specific amplification of the target band. This may be related to the fact that there is less host RNA but considerably more avian HEV RNA in the feces sample (12, 31). It is useful for choosing the sample of the infected birds to detect the virus, and even some other reports also found that the bile is also a good source (20).

According to the results of phylogenetic analysis and genetic distances, avian HEV is defined into four genotypes, inconsistent with five genotypes in the previous study reported by Liu (10). Based on the mean inter genetic distances between genotypes, it seems reasonable to infer that the Genotypes 3 and 4 isolates reported by Liu from Europe, China, and Hungary are all placed in Genotype 3 (Supplementary Tables 5, 6). There is currently not sufficient intra-genotype genetic variation to allow these isolates to be separated into two different genotypes. Our isolate GX20A1 belongs to Genotype 3 and has the highest similarity of 94.1% with CaHEV-GDSZ01 isolated in Guangdong, China. Interestingly, only the sequence of a 339-bp fragment (N7, encoding the helicase gene) in ORF1 could represent the whole genome sequences of avian HEV for classification since 4 out of the 6 datasets (N2, N5, N6, and N7) used in the phylogenetic analysis were consistent with that based on the full-genome sequence (Supplementary Figure 3). This may have advantages over the analysis with the full-genome sequence regarding the easiness, timeliness, and expense that is useful in the analysis and classification of the new isolate in the easy and precise way.

Bayesian analysis showed that the time to most recent common ancestor (tMRCA) of avian HEV was 1,842 (95% *CI*: 1,670–1962), of which the tMRCA of genotypes 1, 2, and 3 were 1,958 (95% *CI*: 1,903–1,983), 1928 (95% *CI*: 1,812–1,987), and 1,937 (95% *CI*: 1,831–1,989), respectively. It is worth noting that the Hungarian strain is at the lowest node of Genotype 3, indicating that Genotype 3 isolates were most likely to spread from Hungary to other places (e.g., China). It has been reported that Chinese breeders were introduced from Hungary at the end of the twentieth century (32). The phylogeographic tree found that isolates GX20A1 and CaHEV-GDSZ01 have the same source. Since it is generally known that there is frequent live poultry trade between Guangdong and Guangxi (21, 33). Therefore, we speculate that isolate GX20A1 was most likely brought in Guangxi from Guangdong by the live poultry trade. Guangxi is also a significant province with the largest amounts of breeding and production of Yellow-chicken in China (33). Besides, it is common with the multi-breed farming, especially in the small-scale farms, and the transmission between different flocks of chickens within the same farm would be possible. So the multi-breed farms, especially ones that have links of newly introduced breeds, need to strengthen the biosecurity isolation, and quarantine measures of the newly introduced birds to avoid the possible spread of viruses to other breeds.

Avian HEV isolate from Donglan Black chicken was first identified in this study, and it was also confirmed that it was a potential susceptible host of avian HEV. Besides, avian HEV has been isolated from wild birds, such as young egrets, kestrels and sparrows, and the transmission of droppings from wild birds has not been ruled out (34, 35). In addition, BSP reflected that the genetic diversity of avian HEV isolates has been low in recent years, and there is not enough evidence to support that avian HEV is rapidly evolving. In the study, one positive selection site in ORF1, at residuals 593 was found. Positive selection site was predicted primarily in the hypervariable hinge region flanking the protease and X domains in ORF1. Previous studies showed that this region might be responsible for differences in replication efficiency (36). We further speculate that the positive selection site in this region might affect viral fitness and influence the pathogenesis of the avian HEV infections (8).

In conclusion, the first avian HEV isolate GX20A1 from Donglan Black chicken was identified. Avian HEV was uniformly defined into four genotypes and the isolate GX20A1 belongs to Genotype 3. The sequences of a 339-bp fragment in ORF1, could be used for the classification of avian HEV. Bayesian analysis showed that Genotype 3 likely originated in the 1930s and isolate GX20A1 shared the same common ancestor with the Guangdong strain CaHEV-GDSZ01. It is essential to perform routine monitoring of the live poultry trade and strengthen the biosecurity isolation and quarantine measures to reduce the risk of inter-provincial and/or cross-breed spread of avian HEV.

## Data Availability Statement

The original contributions presented in the study are included in the article/Supplementary Material, further inquiries can be directed to the corresponding author.

## Ethics Statement

The study focused on avian HEV using a modern molecular approach. The study was approved by the Animal Welfare and the Animal Experimental Ethical Committee of Guangxi University.

## Author Contributions

FF and QD completed data analysis and the draft and contributed to the experiment. QL, WZ, and JG assisted in this experiment. PW provided the funding of research, reviewed, and approved the final manuscript. All authors contributed to the article and approved the submitted version.

## Funding

This work was supported by the Guangxi Program for Modern Agricultural Industry Technical System Construction-Chicken Industry (nycytxgxcxtd-19-03) and the Guangxi Special Funding on Science and Technology Research (AA17204057).

## Conflict of Interest

The authors declare that the research was conducted in the absence of any commercial or financial relationships that could be construed as a potential conflict of interest.

## Publisher's Note

All claims expressed in this article are solely those of the authors and do not necessarily represent those of their affiliated organizations, or those of the publisher, the editors and the reviewers. Any product that may be evaluated in this article, or claim that may be made by its manufacturer, is not guaranteed or endorsed by the publisher.
