# Supplementary material for: Emergence and Molecular Characterization of an Avian Hepatitis E Virus From Donglan Black Chicken in Southern China
Source: Front Vet Sci. 2022 May 26;9:901292. doi: 10.3389/fvets.2022.901292 (PMC9469092; doi:10.3389/fvets.2022.901292)
Supplement: Supplementary file 1 [file Table_1.docx]

Supplementary Material

| **Supplementary Table 1 Primer sequences used for detection or amplification of the avian HEV** | | | |
| --- | --- | --- | --- |
| Primers | Sequences (5'→3') | Positions | Product length (bp) |
| F9F^a^ | AATGGTAGCTCCGTGGTTTGGTATGC | 6252-6277 | 385 |
| aHCGR^a^ | ACTATGCCCGAGATGGGAGG | 6615-6634 |  |
|  |  |  |  |
| AHEV ORF2/ F-1/SD^b^ | TCGCCYGGTAAYACWAATGC | 5457-5476 | 278 |
| AHEV ORF2/R-1/SD^b^ | GCGTTSCCSACAGGYCGGCC | 5715-5734 |  |
|  |  |  |  |
| AHEV ORF2/F-2/SD^b^ | ACWAATGCYAGGGTCACCCG | 5469-5489 | 242 |
| AHEV ORF2/R-2/SD^b^ | ATGTACTGRCCRCTSGCCGC | 5691-5710 |  |
| ^a^ The primer sequence is derived from Iqbal *et al* (1). | | | |
| ^b^ The primer sequence is derived from Sun *et al* (2). | | | |

| **Supplementary Table 2 Primer sequences used for detection or amplification of ALV, MDV and REV isolates** | | | |
| --- | --- | --- | --- |
| Primers | Sequences (5'→3') | Tm (℃) | Product length (bp) |
| ALV-p27 Forward | GGATGAGGTGACTAAGAAAG | 55 | 545 |
| ALV-p27 Reverse | CGAACCAAAGGTAACACAC |  |  |
|  |  |  |  |
| MDV-*meq* Forward | CCGTCTAGAAGGCGGGCACGGTAC | 60 | 1095 |
| MDV-*meq* Reverse | CGGAAGCTTAAACATGGGGCATAGACG |  |  |
|  |  |  |  |
| REV-*LTR* Forward | CATACTGGAGCCAATGGT | 50 | 291 |
| REV-*LTR* Reverse | AATGTTGTAGCGAAGTACT |  |  |
|  |  |  |  |
| REV-*env* Forward | CAGGAATTCAAGAATGGACTGTCTCAC | 58 | 1000 |
| REV-*env* Reverse | ATTGTCGACCTGGTGGAGGACATAGC |  |  |
|  |  |  |  |
| FAdV-4 Forward | AACGTCAATCCCTTCAACCACC | 60 | 1319 |
| FAdV-4 Reverse | TTGCCTGTGGCGAAAGGCG |  |  |

| **Supplementary Table 3 Primer sequences used for avian HEV genome amplification** | | | |
| --- | --- | --- | --- |
| Primers | Sequences (5'→3') | Positions in the genome | Product length(bp) |
| AHEV_F1-1^a^ | CCATGCCAGGGTAAGAATG | -14 |  |
| AHEV_R1-1^a^ | AAAACAGCAAGGACCTCC | 1859-1876 |  |
| AHEV_F1-2^a^ | CCAGGGTAAGAATGGACG | 1-18 | 1311 |
| AHEV_R1-2^a^ | TAATCCAGGTGGCGAGC | 1295-1311 |  |
| AHEV_F2^b^ | TTTTGGTTCAAGGTTTGGGAG | 1221-1241 | 1197 |
| AHEV_R2^b^ | AACCCCTTTCACCAATTCACTG | 2396-2417 |  |
| AHEV_F3-1^a^ | GGCTGTGTGGCATGTTCCA | 1945-1981 |  |
| AHEV_R3-1^a^ | GGTAAAGAGCCACCATCCAAT | 3988-4008 |  |
| AHEV_F3-2^a^ | CCGTGATGGTGACTTGTTGGTTGT | 2240-2263 | 1343 |
| AHEV_R3-2^a^ | GGCACATCTCCGCATACTC | 3564-3582 |  |
| AHEV_F4-1^a^ | CCCTTCAACATTGGAGTATGC | 3551-3571 |  |
| AHEV_R4-1^a^ | ATCTGGTACCGTGCGAGT | 4877-4894 |  |
| AHEV_F4-2^a^ | ACATTGGAGTATGCGGAGATG | 3558-3578 | 1259 |
| AHEV_R4-2^a^ | TTGAGCGCTCCACTGGGCT | 4798-4816 |  |
| AHEV_F5^b^ | GCGCGTGAGACTTTAACAAC | 4542-4561 | 1100 |
| AHEV_R5^b^ | GAACAAAACACCGAGAATGC | 5622-5641 |  |
| AHEV_F6^b^ | AGGCGTGGCTGAAGATGG | 5590-5607 | 1044 |
| AHEV_R6^b^ | ACACTGCCCGAGATGGGAGG | 6650-6633 |  |
| ^a^ The primer sequence is derived from Zhao *et al*(3). | | | |
| ^b^ Primers were designed according to the sequences of the avian HEV strains CaHEV-GDSZ01 (MK050107)and CaHEV (GU954430). | | | |

| **Supplementary Table 4** Details of the Avian HEV reference isolates used in this study | | | | | |
| --- | --- | --- | --- | --- | --- |
| No. | Strains | Accession number | Country of origin | Years | Genotype |
| 1 | AaHEV | AM943647 | Australia | 1986 | 1 |
| 2 | HH-F9 | JN597006 | South Korea | 2011 | 1 |
| 3 | JY-F2 | KC454286 | South Korea | 2011 | 1 |
| 4 | Avirulent | EF206691 | USA | 2004 | 2 |
| 5 | AY535004 | AY535004 | USA | 2001 | 2 |
| 6 | GI-B | KM377618 | South Korea | 2014 | 2 |
| 7 | VaHEV-HB | MG976720 | Hebei, China | 2017 | 2 |
| 8 | 121 | MH507048 | Shandong, China | 2018 | 2 |
| 9 | 65 | MH507047 | Shandong, China | 2018 | 2 |
| 10 | 68 | MH507046 | Shandong, China | 2018 | 2 |
| 11 | 42 | MH507045 | Shandong, China | 2018 | 2 |
| 12 | 18-PL514 | MH636899 | Poland | 2018 | 2 |
| 13 | 18-PL513 | MH636898 | Poland | 2018 | 2 |
| 14 | 18-PL284 | MH636897 | Poland | 2018 | 2 |
| 15 | 18-PL243 | MH636895 | Poland | 2018 | 2 |
| 16 | 17-PL19 | MH636893 | Poland | 2017 | 2 |
| 17 | 17-PL18 | MH636892 | Poland | 2017 | 2 |
| 18 | 17-PL14A | MH636890 | Poland | 2017 | 2 |
| 19 | 17-PL9 | MH636886 | Poland | 2017 | 2 |
| 20 | 17-PL8 | MH636885 | Poland | 2017 | 2 |
| 21 | 17-PL3B | MH636883 | Poland | 2017 | 2 |
| 22 | aHEVSP-425 | EU919192 | Spain | 2008 | 2 |
| 23 | aHEVSP-417 | EU919191 | Spain | 2008 | 2 |
| 24 | aHEVSP-291 | EU919190 | Spain | 2007 | 2 |
| 25 | aHEVSP-64 | EU919188 | Spain | 2007 | 2 |
| 26 | F3-17 | KJ495803 | USA | 2013 | 2 |
| 27 | F2-6 | KJ495802 | USA | 2013 | 2 |
| 28 | F3-14 | KJ495801 | USA | 2013 | 2 |
| 29 | F1-62 | KJ495799 | USA | 2013 | 2 |
| 30 | F7-50 | KJ495798 | USA | 2013 | 2 |
| 31 | F4-26 | KJ495797 | USA | 2013 | 2 |
| 32 | F1-59 | KJ495796 | USA | 2013 | 2 |
| 33 | F7-51 | KJ495794 | USA | 2013 | 2 |
| 34 | F4-30 | KJ495793 | USA | 2013 | 2 |
| 35 | F2-2 | KJ495792 | USA | 2013 | 2 |
| 36 | F2-8 | KJ495791 | USA | 2013 | 2 |
| 37 | W838-14 | MW589652 | Australia | 2014 | 2 |
| 38 | W865-14 | MW589651 | Australia | 2014 | 2 |
| 39 | EaHEV | AM943646 | Hungary | 2005 | 3 |
| 40 | CaHEV | GU954430 | Shandong, China | 2009 | 3 |
| 41 | HU-16773-2010 | JN997392 | Hungary | 2010 | 3 |
| 42 | TWNaHEV | KF511797 | Taiwan, China | 2012 | 3 |
| 43 | CaHEV-GDSZ01 | MK050107 | Guangdong, China | 2018 | 3 |
| 44 | CHN-SN-C1 | MG922665 | Shaanxi, China | 2017 | 3 |
| 45 | CHN-SN-D1 | MG922666 | Shaanxi, China | 2017 | 3 |
| 46 | CHN-SN-G1 | MG922667 | Shaanxi, China | 2017 | 3 |
| 47 | CHN-SN-R1 | MG922668 | Shaanxi, China | 2017 | 3 |
| 48 | YT-aHEV | MW924815 | Shandong, China | 2020 | 3 |
| 49 | SilkieHEV | MN562265 | Gansu, China | 2018 | 4 |

| Supplementary Table 5 The mean inter-genotypes distance between the genotypes | | | | |
| --- | --- | --- | --- | --- |
|  | **Genotype 1** | **Genotype 2** | **Genotype 3** | **Genotype 4** |
| Genotype 1 |  |  |  |  |
| Genotype 2 | 0.213 |  |  |  |
| Genotype 3 | 0.212 | 0.215 |  |  |
| Genotype 4 | 0.227 | 0.236 | 0.225 |  |
| The number of base substitutions per site from averaging over all sequence pairs between genotypes are shown. All results are based on the pairwise analysis of 14 sequences and the number of sequences analyzed per groups was: Genotype 1, n = 3; Genotype 2, n =6; Genotype 3, n = 7 and Genotype 4, n = 1. Analyses were conducted using the Maximum Composite Likelihood model. The rate variation among sites was modeled with a gamma distribution (shape parameter = 1). Evolutionary analyses were conducted in MEGA X. | | | | |

| **Supplementary Table 6 The mean inter-genotypes distance between 5 genotypes** | | | | | |
| --- | --- | --- | --- | --- | --- |
|  | **Genotype 1** | **Genotype 2** | **Genotype 3** | **Genotype 4** | **Genotype 5** |
| **Genotype 1** |  |  |  |  |  |
| **Genotype 2** | 0.213 |  |  |  |  |
| **Genotype 3** | 0.212 | 0.215 |  |  |  |
| **Genotype 4** | 0.213 | 0.213 | **0.181** |  |  |
| **Genotype 5** | 0.227 | 0.236 | 0.226 | 0.222 |  |
| The number of base substitutions per site from averaging over all sequence pairs between genotypes is shown. All results are based on the pairwise analysis of 14 sequences, and the number of sequences analyzed per group was: Genotype 1, n = 3; Genotype 2, n =6; Genotype 3, n = 5; Genotype 4, n = 2 and Genotype 5, n = 1. Analyses were conducted using the Maximum Composite Likelihood model. The rate variation among sites was modeled with a gamma distribution (shape parameter = 1). Evolutionary analyses were conducted in MEGA X. | | | | | |


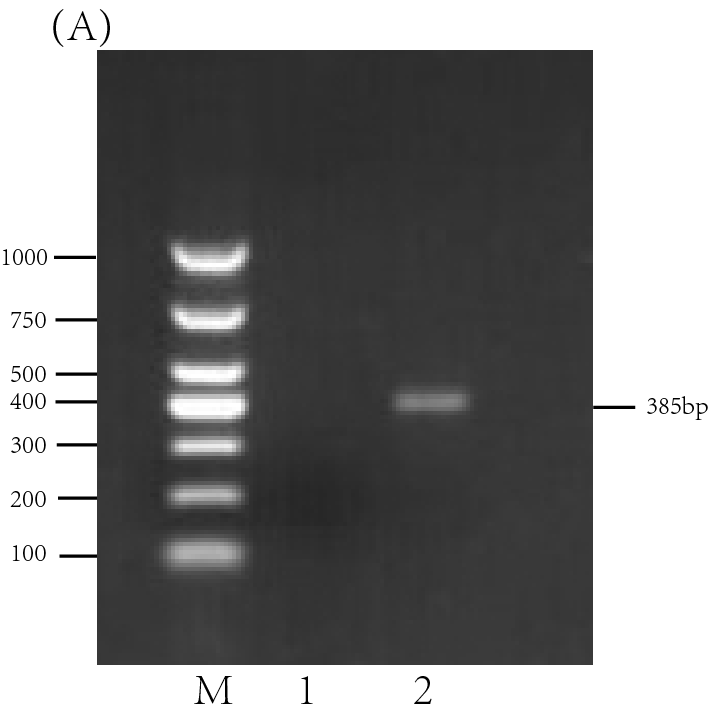

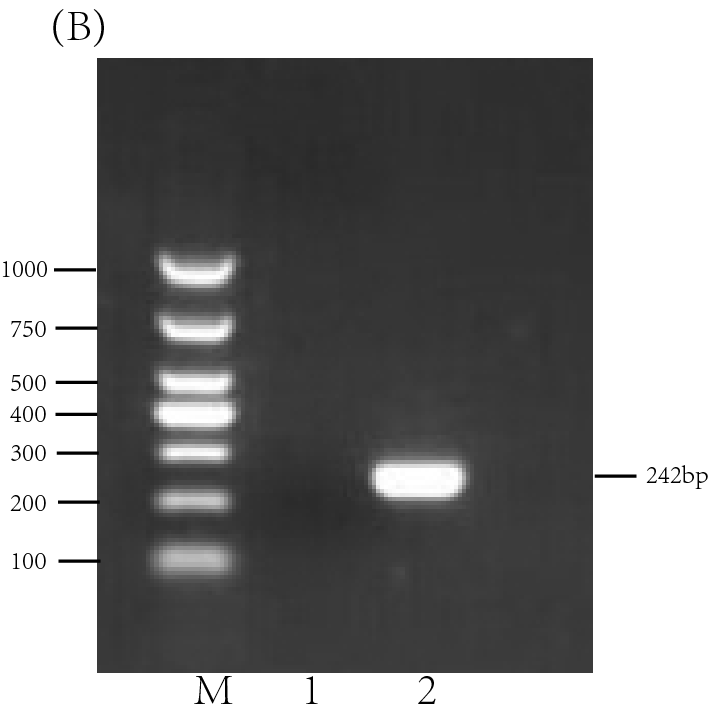


**Supplementary Figure 1** **Results of PCR identification.** (A) Electrophoretic patterns of PCR products with primers of F9F and aHCGR; (B) Electrophoretic patterns of PCR products with primers of AHEV ORF2/F-2/SD and AHEV ORF2/R-2/SD; Line M, DL1000 Marker; Line 1, Negative control; Line 2, Sample.

**
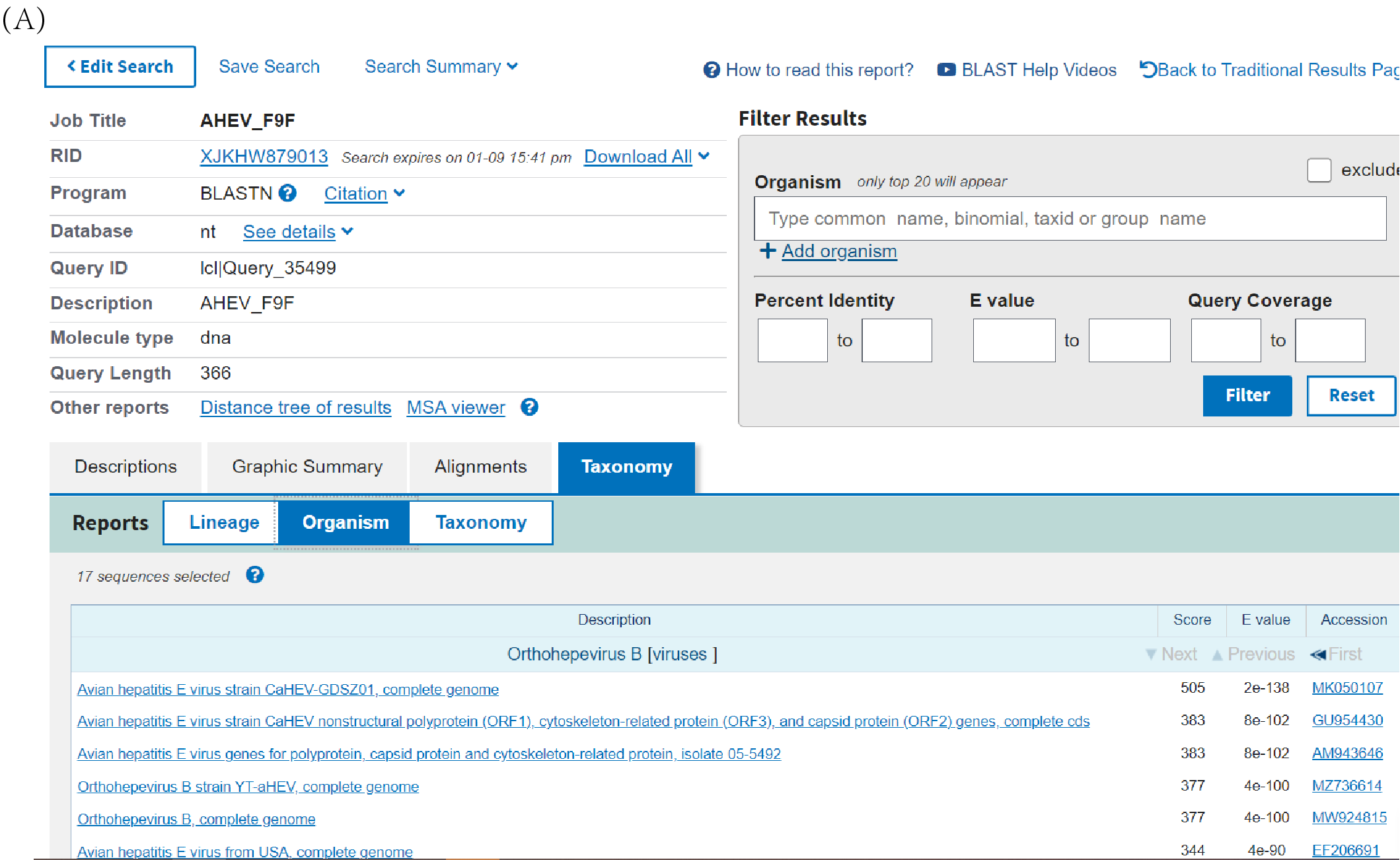
**


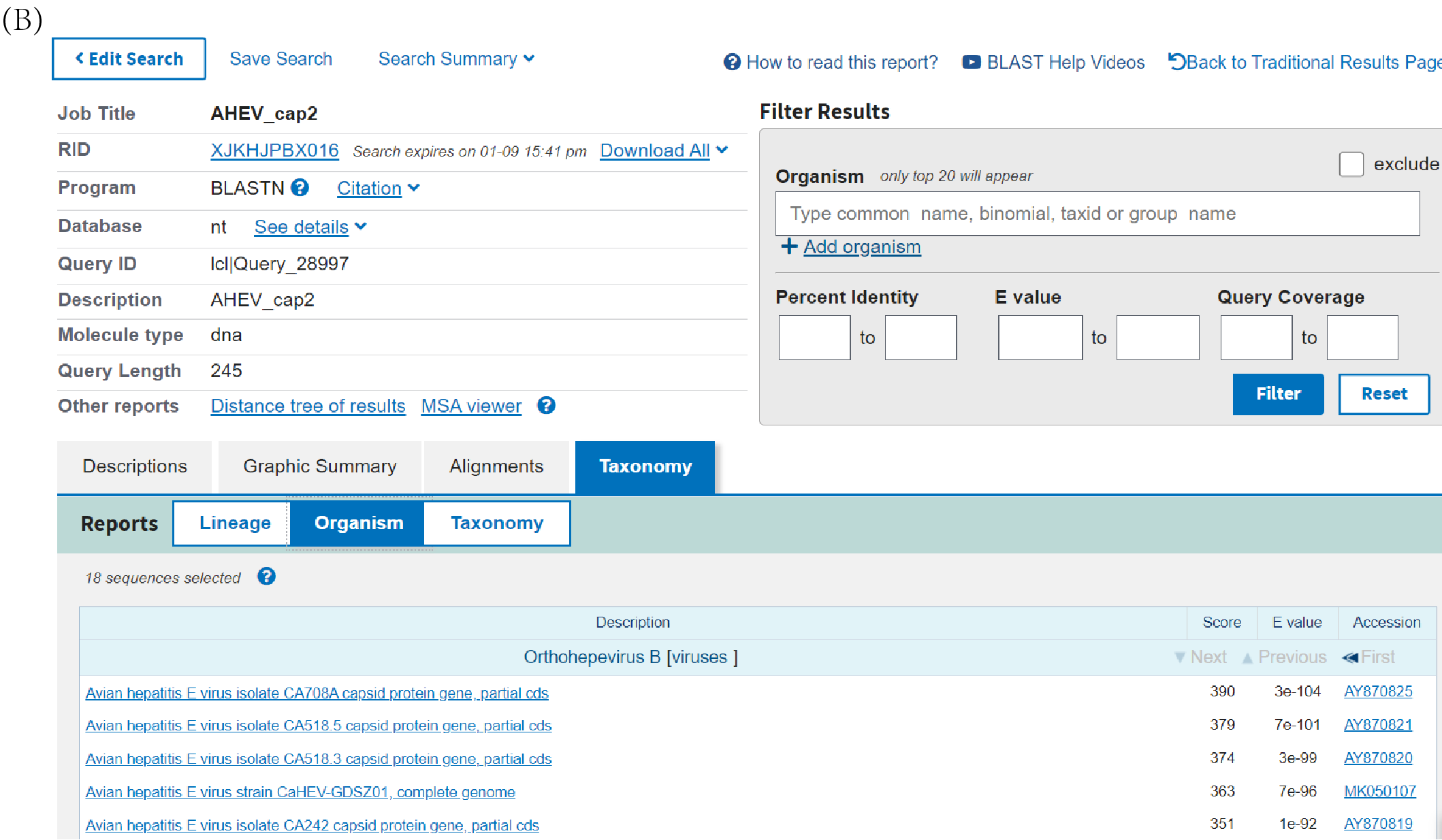


**Supplementary Figure 2** **BLAST results.** (A) BLAST result of the sequence (primers of F9F and aHCGR); (B) BLAST result of the sequence (primers of AHEV ORF2/F-2/SD and AHEV ORF2/R-2/SD)

**
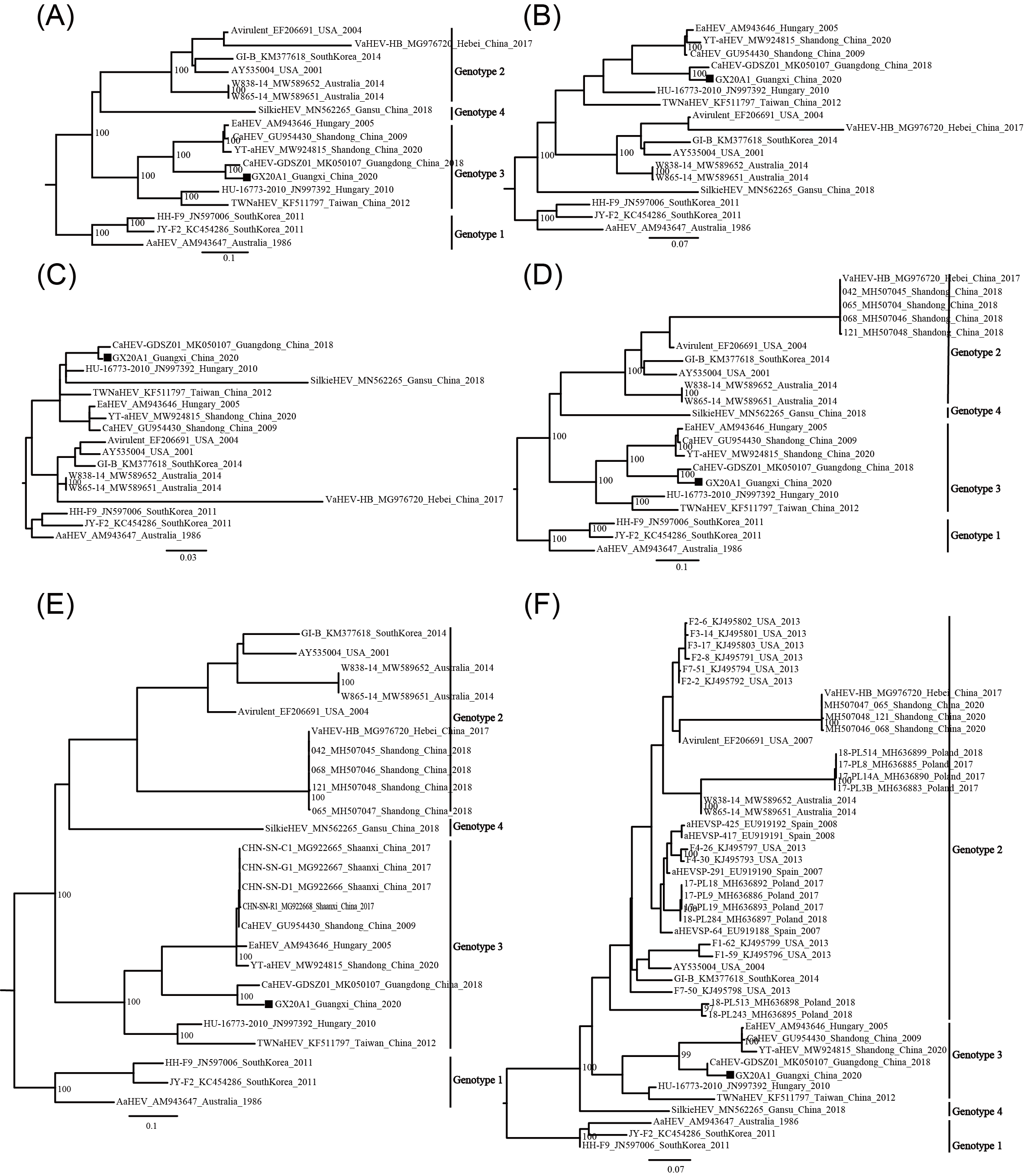
**

**Supplementary Figure 3** **Phylogenetic analysis by the 3 ORFs and different fragment in ORF1 of avian HEV.** The trees were constructed based on the maximum likelihood (ML) method implemented by IQ-TREE v1.6.12. Solid black square indicates the isolate in this study. (A) ORF1 phylogenetic tree (N2=17); (B) ORF2 phylogenetic tree (N3=17); (C) ORF3 phylogenetic tree (N4=17); (D) phylogenetic tree of 2755 bp in ORF1 (N5=21, 177-2922nt); (E) phylogenetic tree of 570 bp in ORF1 (N6=25, 1595-2157 nt); (F) phylogenetic tree of 339 bp in ORF1 (N7=45, 2584-2922 nt).


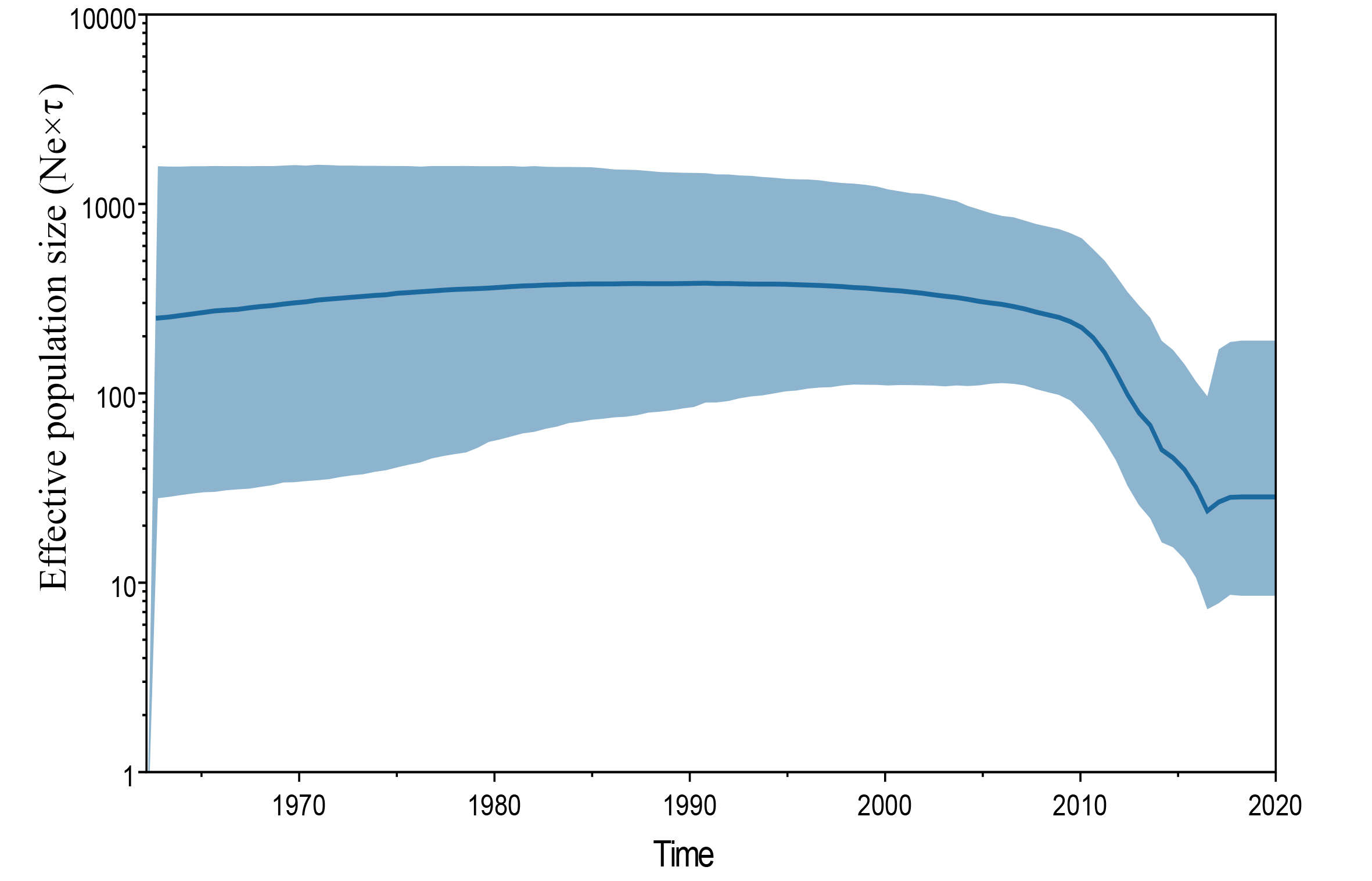


**Supplementary Figure 4 Bayesian skyline plot (BSP) obtained by analyzing the 45 avian HEV isolates sampled at different times.** Ordinate: the effective number of infections (*N_e_*) multiplied by mean viral generation time (*τ*); Abscissa: Time. Dark blue line indicates the mean value of genetic diversity, and the light blue shading shows the 95% confidence interval.

**REFERENCES**

1. Iqbal T, Rashid U, Idrees M, Afroz A, Kamili S, Purdy MA. A Novel Avian Isolate of Hepatitis E Virus from Pakistan. *Virol J* (2019) 16(1):142. Epub 2019/11/23. doi: 10.1186/s12985-019-1247-0.

2. Sun ZF, Larsen CT, Dunlop A, Huang FF, Pierson FW, Toth TE, et al. Genetic Identification of Avian Hepatitis E Virus (HEV) from Healthy Chicken Flocks and Characterization of the Capsid Gene of 14 Avian Hev Isolates from Chickens with Hepatitis-Splenomegaly Syndrome in Different Geographical Regions of the United States. *J Gen Virol* (2004) 85(Pt 3):693-700. Epub 2004/03/03. doi: 10.1099/vir.0.19582-0.

3. Zhao Q, Zhou EM, Dong SW, Qiu HK, Zhang L, Hu SB, et al. Analysis of Avian Hepatitis E Virus from Chickens, China. *Emerg Infect Dis* (2010) 16(9):1469-72. Epub 2010/08/26. doi: 10.3201/eid1609.100626.
